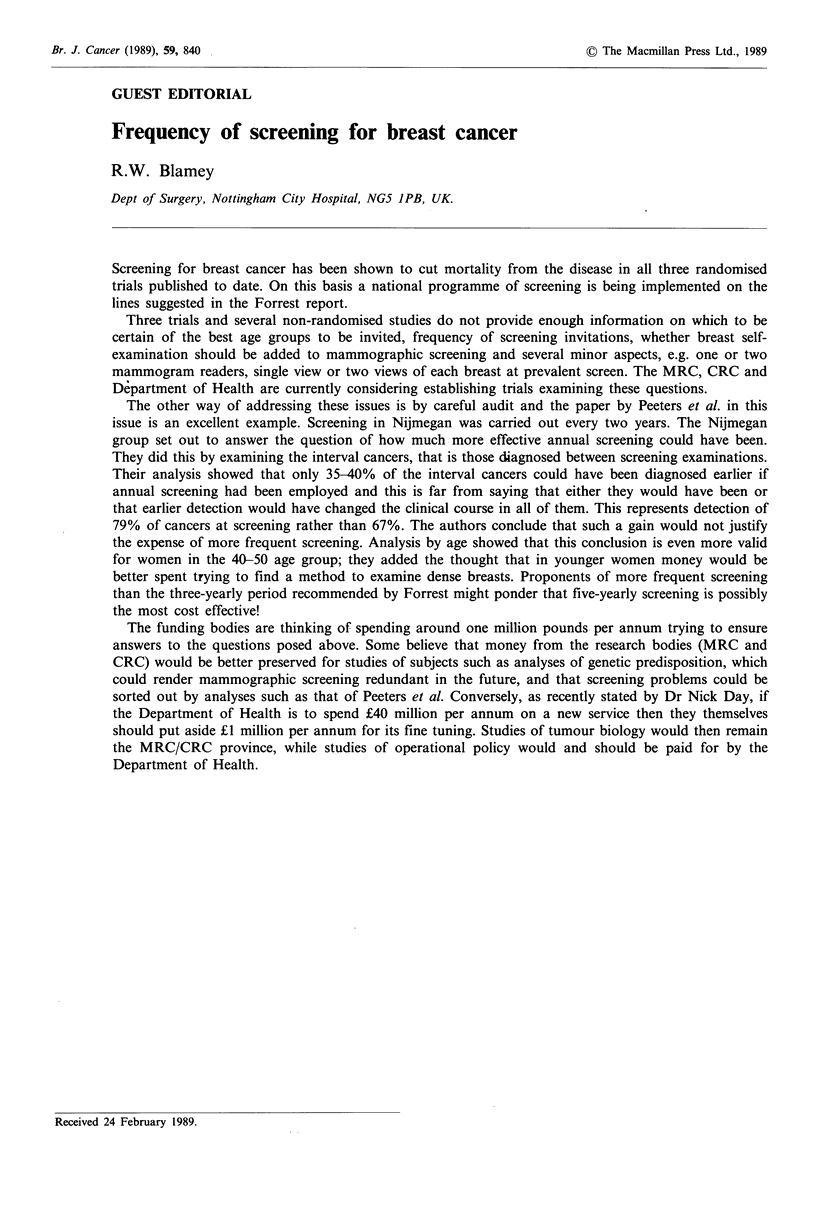# Frequency of screening for breast cancer

**Published:** 1989-06

**Authors:** R.W. Blamey


*Br. J. Cancer* (1989), **59**, 840 © The Macmillan Press Ltd., 1989

GUEST EDITORIAL

### Frequency of screening for breast cancer

R.W. Blamey

*Dept of Surgery, Nottingham City Hospital, NG5 1PB, UK.*

Screening for breast cancer has been shown to cut mortality from the disease in all three randomised trials published to date. On this basis a national programme of screening is being implemented on the lines suggested in the Forrest report.

Three trials and several non-randomised studies do not provide enough information on which to be certain of the best age groups to be invited, frequency of screening invitations, whether breast self-examination should be added to mammographic screening and several minor aspects, e.g. one or two mammogram readers, single view or two views of each breast at prevalent screen. The MRC, CRC and Department of Health are currently considering establishing trials examining these questions.

The other way of addressing these issues is by careful audit and the paper by Peeters *et al.* in this issue is an excellent example. Screening in Nijmegan was carried out every two years. The Nijmegan group set out to answer the question of how much more effective annual screening could have been. They did this by examining the interval cancers, that is those diagnosed between screening examinations. Their analysis showed that only 35–40% of the interval cancers could have been diagnosed earlier if annual screening had been employed and this is far from saying that either they would have been or that earlier detection would have changed the clinical course in all of them. This represents detection of 79% of cancers at screening rather than 67%. The authors conclude that such a gain would not justify the expense of more frequent screening. Analysis by age showed that this conclusion is even more valid for women in the 40–50 age group; they added the thought that in younger women money would be better spent trying to find a method to examine dense breasts. Proponents of more frequent screening than the three-yearly period recommended by Forrest might ponder that five-yearly screening is possibly the most cost effective!

The funding bodies are thinking of spending around one million pounds per annum trying to ensure answers to the questions posed above. Some believe that money from the research bodies (MRC and CRC) would be better preserved for studies of subjects such as analyses of genetic predisposition, which could render mammographic screening redundant in the future, and that screening problems could be sorted out by analyses such as that of Peeters *et al.* Conversely, as recently stated by Dr Nick Day, if the Department of Health is to spend £40 million per annum on a new service then they themselves should put aside £1 million per annum for its fine tuning. Studies of tumour biology would then remain the MRC/CRC province, while studies of operational policy would and should be paid for by the Department of Health.

Received 24 February 1989.